# The effect prophylactic internal iliac artery balloon occlusion in patients with placenta previa or placental accreta spectrum: a systematic review and meta‐analysis

**DOI:** 10.1186/s12958-021-00722-3

**Published:** 2021-03-04

**Authors:** Anisodowleh Nankali, Nader Salari, Mohsen Kazeminia, Masoud Mohammadi, Samira Rasoulinya, Melika Hosseinian-Far

**Affiliations:** 1 Department of Obstetrics and Gynecology, School of Medicine, Kermanshah University of Medical Sciences, Kermanshah, Iran; 2 Department of Biostatistics, School of Health, Kermanshah University of Medical Sciences, Kermanshah, Iran; 3 Student Research Committee, Kermanshah University of Medical Sciences, Kermanshah, Iran; 4 Department of Nursing, School of Nursing and Midwifery, Kermanshah University of Medical Sciences, Kermanshah, Iran; 5 Department of Food Science & Technology, Faculty of Agriculture, Ferdowsi University of Mashhad (FUM), Mashhad, Iran

**Keywords:** Internal iliac artery balloon, Placenta previa, Placenta accreta spectrum, Systematic review and meta‐analysis

## Abstract

**Background:**

Placenta previa describes a placenta that extends partially or completely over the internal cervical oss. Placenta previa is one of the leading causes of widespread postpartum hemorrhage and maternal mortality worldwide. Another cause of bleeding in pregnant women is Placenta accreta spectrum. Therefore, the aim of the present systematic review and meta-analysis is to determine the effect of prophylactic balloon occlusion of the internal iliac arteries in patients with placenta previa or placental accreta spectrum (PAS).

**Methods:**

In this systematic review and meta-analysis, to identify and select relevant studies, the SID, MagIran, ScienceDirect, Embase, Scopus, PubMed, Web of Science, and Google Scholar databases were searched, using the keywords of internal iliac artery balloon, placenta, previa, balloon, accreta, increta and percreta, without a lower time limit and until 2020. The heterogeneity of the studies was examined using the I^2^ index, and subsequently a random effects model was applied. Data analysis was performed within the Comprehensive Meta-Analysis software (version 2).

**Results:**

In the review of 29 articles with a total sample size of 1140 in the control group, and 1225 in the balloon occlusion group, the mean difference between the two groups was calculated in terms of Intraoperative blood loss index (mL) and it was derived as 3.21 ± 0.38; moreover, in 15 studies with a sample size of 887 in the control group, and 760 in the balloon occlusion group, the mean difference between the two groups in terms of gestation index (weeks) was found as 2.84 ± 0.49; and also with regards to hysterectomy balloon occlusion after prophylactic closure of the iliac artery, hysterectomy (%) balloon occlusion was calculated as 8.9 %, and this, in the hysterectomy control group (%) was obtained as 31.2 %; these differences were statistically significant and showed a positive effect of the intervention (*P* < 0.05).

**Conclusion:**

The results of this study show that the use of prophylactic internal iliac artery balloon occlusion in patients with placenta previa or Placenta accreta spectrum has benefits such as reduced intraoperative blood loss, reduced hysterectomy and increased gestation (weeks), which can be considered by midwives and obstetricians.

## Background

Placenta previa is a type of placenta that extends partially or completely over the internal cervical oss [1]. Placenta previa is one of the leading causes of widespread postpartum hemorrhage and maternal mortality worldwide [2]. The prevalence of placenta previa in women is 0.56 % [3]. The risk of hysterectomy after cesarean delivery in the case of placenta previa is 3.5 %, which is higher compared to mothers without it. Increasing the rate of cesarean delivery due to placenta previa has a significant effect on the cost of gynecological medical care [4]. The main cause of placenta previa is unknown. Maternal age, previous cesarean section, abortion, uterine myoma, high body mass index, high infant weight, male fetal, maternal tobacco use, and history of uterine surgery are among the predisposing factors of this disease [5].

Another cause of bleeding in pregnant women is placental adhesions. placental adhesions is an abnormal invasion of the placenta to the uterine wall and is classified as accreta, percreta, or increta depending on the degree of invasion of the myometrium [5, 6]. The prevalence of placental accreta spectrum in the third trimester in pregnant women is between 0.3 and 2 %; this is a life-threatening condition and approximately 90 % of these patients require blood transfusion [7, 8]. In many countries around the world, cesarean section is a growing trend that has increased cases of placental adhesions, which in turn has increases concerns [7].

Establishing homeostasis in placenta requires the use of surgical techniques. These methods include multiple placement of hemostatic sutures in the placental bed, compression sutures of the uterus with or without intrauterine balloon tamponade, use of FLOSEAL in the placental bed, and uterin artery lrgation [9–13]. If these surgical procedures to control bleeding are unsuccessful, hysterectomy is the usual solution to save the patient’s life [14]. However, extensive bleeding often makes surgical treatment even more challenging. Therefore, preventive measures to reduce bleeding can be beneficial [9]. Recently, preoperative placement of internal iliac artery catheters with intraoperative arterial balloon occlusion has become a common preventive procedure [15].

Collapsed intra-arterial balloons do not completely block blood flow to the uterus, yet they lower blood pressure to the site of obstruction. By reducing the amount of bleeding, hemostatic methods can be more practical [16–22]. Obstructive balloon occlusion was first described by Dubois et al. as, and has since led to dissimilar outcomes in patients with adhesions, making it a controversial procedure [23]. Nonetheless, the advantages of this method include reducing bleeding in the area of surgery to achieve homeostasis or hysterectomy [24].

Considering the importance of reducing bleeding due to placenta previa and Placenta accreta spectrum to reduce their mortality among pregnant women, and also due to the inconsistent results from research in this area, this systematic review and meta-analysis aimed to determine the effect prophylactic internal iliac artery balloon occlusion in patients with placenta previa or Placenta accreta spectrum.

## Methods

In this meta-analysis, the SID, MagIran, ScienceDirect, Embase, Scopus, PubMed, Web of Science and Google Scholar databases were searched to identify and select relevant studies. The keywords used were: internal iliac artery balloon, placenta, previa, balloon, accreta, increta, and percreta, and all possible combinations of these words. No time constraints were considered in the search. The information of the identified articles was transferred into the EndNote bibliography management software (EndNote X8). In order to maximize the comprehensiveness of the search, the lists of references used within all identified articles were manually reviewed.

### Inclusion criteria

Studies that examined the effect prophylactic internal iliac artery balloon occlusion in patients with placenta previa or Placenta accreta spectrum, interventional studies, and studies for which a full text was available.

### Exclusion criteria

Unrelated studies, studies without sufficient data, duplicate papers, studies with an unclear methodology.

### Study selection

Initially, duplicate studies in various databases were excluded from this study, and only one copy was retained. Then a list of the titles of all the remaining articles was prepared, for further analysis. In the first stage, screening, the title and abstract of the remaining articles were carefully examined, and a number of articles were removed considering the inclusion and exclusion criteria. In the second stage, i.e. eligibility evaluation, the full text of the studies, remaining from the screening stage, were thoroughly examined according to the criteria, and similarly, a number of other unrelated studies were excluded. To prevent subjectivity, article review, and data extraction activities were performed by two reviewers, independently. If an article was not included, the reason for excluding it was mentioned. In cases where there was a disagreement between the two reviewers, a third person reviewed the article.

### Quality evaluation

In order to examine the quality of the remaining articles (i.e. methodological validity and results), a checklist appropriate to the type of study was adopted. STROBE checklists are commonly used to critique and evaluate the quality of observational studies. The checklist consists of six scales/general sections that are: title, abstract, introduction, methods, results, and discussion. Some of these scales have subscales, resulting in a total of 32 fields (subscales). In fact, these 32 fields represent different methodological aspects of a piece of research. Examples of subscales include title, problem statement, study objectives, study type, statistical population, sampling method, sample size, the definition of variables and procedures, data collection method(s), statistical analysis techniques, and findings. Accordingly, the maximum score that can be obtained during the quality evaluation phase and using the STROBE checklist is 32. By considering the score of 16 as the cut-off point [25], any article with a score of 16 or above is considered as a medium or a high-quality article.

### Data extraction

Information on all final articles was extracted from a different pre-prepared checklist. This checklist includes article title, first author’s name, year of publication, sample size, place of study, maternal age (years), intraoperative blood loss (mL), blood transfusion (units), blood transfusion volume (ml), placenta type, and gestation (weeks), Hysterectomy (%).

### Statistical analysis

Frequency and percentage as well as the standardized mean difference indices were used to combine the results of different studies. The I^2^ test was used to evaluate the homogeneity between studies, and due to the found heterogeneity in the studies, a random effects model was used to combine the studies and perform the meta-analysis. When the I^2^ index is less than 25 %, heterogeneity was considered as low; between 25 and 275 %, this was considered as moderate heterogeneity, and more than 75 % as high heterogeneity. P value less than 0.05 was considered significant. The Egger’s test and corresponding funnel plots were also used to examine the publication bias. Data analysis was performed within the Comprehensive Meta-Analysis software (version 2).

## Results

**Fig. 1:**
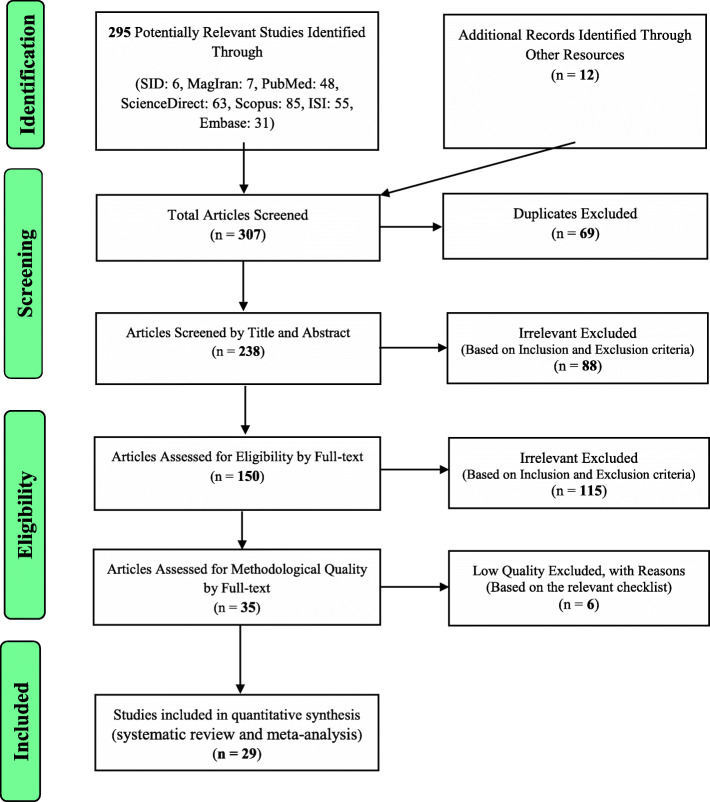
The flowchart on the stages of including the studies in the systematic review and meta-analysis (PRISMA 2009)

The study selection process was conducted according to the Preferred Reporting Items for Systematic Reviews and Meta-Analyses (PRISMA) guidelines. Based on the initial search in the database, 295 possible related articles were identified and transferred into the EndNote bibliography management software. Moreover, 12 further studies were added after reviewing the lists of references of the identified articles. Out of a total of 307 identified studies, 69 studies were duplicates and were therefore excluded. In the screening phase, out of the remaining 238 studies, 88 articles were removed after examining their titles and abstracts, and based on the inclusion and exclusion criteria. In the eligibility evalaution stage, out of the remaining 150 studies, 115 irrelevant articles were removed by studying the full text of the articles and in accordance with the inclusion and exclusion criteria. In the quality evaluation stage, by reading the full text of the article and based on the score obtained from the STROBE checklist, out of the remaining 35 studies, 6 low quality studies were omitted. Finally, 29 articles that were published between 2006 and August 2020 entered the analysis stage (Fig. 1).

The sample size of the balloon occlusion group was 1225, and the sample size in the control group was 1140. The characteristics of the eligible studies included in the systematic review and meta-analysis are provided in Table 1 (Table 1).

**Table 1:** Profile of studies entered into systematic review and meta-analysis

Author, year, Reference	Country	Type placenta	Variable	Balloon occlusion group	Control group	*p*-value
Yu SCH, 2020, [26]	Hong Kong	previa	Maternal age (years)	35.3 [31.5-37.6]	36.6 [32.7-39.1]	-
			Sample size (n)	20	20	-
			Intraoperative blood loss (mL)	1451 [1024-2388]	1454 [888-2300]	0.945
			Blood transfusion (units)	0.5 [0-2.75]	2 [0-4]	0.55
			Gestation (weeks)	36.6 [35.2-37.2]	36.1 [34.8-37.7]	0.968
			Hysterectomy (%)	0.0	0.0	-
Sallam, 2018, [27]	Egypt	previa	Maternal age (years)	29.71±2.84	29.68± 2.53	-
			Sample size (n)	62	62	-
			Intraoperative blood loss (mL)	1151.61±246.38	1800±980	0.0001
			Gestation (weeks)	35.55±0.77	35.58±0.62	0.977
Darwish, 2014, [28]	Egypt	previa	Maternal age (years)	33.8	33.5	-
			Sample size (n)	32	32	-
			Intraoperative blood loss (mL)	1900	2800	0.019
			Blood transfusion (units)	2.9	4.7	0.142
			Gestation (weeks)	35.6	34.3	0.008
Omar, 2017, [29]	Egypt	previa	Maternal age (years)	34.7±6.8	33.1±5.9	-
			Sample size (n)	20	20	-
			Intraoperative blood loss (mL)	1076±545	1664±647	<0.001
			Blood transfusion (units)	2	4	0.13
			Hysterectomy (%)	5	10	0.41
Jiang, 2013, [30]	China	previa	Sample size (n)	13	28	-
			Intraoperative blood loss (mL)	1429±875	4600± 2090	0.000
			Blood transfusion volume (ml)	920±438	3600± 1225	0.000
			Hysterectomy (%)	84.61	89.28	0.670
Broekman, 2015, [31]	Netherlands	previa	Maternal age (years)	32.6 ± 4.3	34.1 ± 3.7	-
			Sample size (n)	42	26	-
			Intraoperative blood loss (mL)	800 (488–1113)	1000 (694–1307)	0.06
			Blood transfusion (units)	4	6	
			Gestation (weeks)	37 (36-38)	37(35-38)	0.16
Mei, 2018, [32]	China	previa	Sample size (n)	20	12	-
			Intraoperative blood loss (mL)	800 (500-1800)	1875 (500-7600)	0.01
			Hysterectomy (%)	0.0	8.3	<0.01
Soyama, 2017, [33]	Germany	previa	Maternal age (years)	35 (27–42)	34 (19–43)	-
			Sample size (n)	50	216	-
			Intraoperative blood loss (mL)	979 (287–2369)	2189 (2133–3360)	<0.01
			Gestation (weeks)	36 (30-38)	37 (30-38)	0.87
Fan, 2017, [34]	China	previa	Maternal age (years)	32.6±0.6	32.0±0.4	-
			Sample size (n)	74	89	-
			Intraoperative blood loss (mL)	1236.0±138.2	1694.0±144.3	0.01
			Blood transfusion volume (ml)	728.0±113.6	1205.0±138.7	0.01
			Gestation (weeks)	36.5±0.1	36.4±0.2	0.65
			Hysterectomy (%)	2.7	5.6	0.36
Dai, 2018, [35]	China	previa	Sample size (n)	42	20	-
			Intraoperative blood loss (mL)	2900.00 ± 2352.21	4549.77 ± 2366.67	0.025
			Blood transfusion (units)	8.40 ± 7.14	13.00 ± 7.93	0.018
			Hysterectomy (%)	2.3	35	0.027
Ono, 2018, [36]	Japan	previa	Maternal age (years)	33.7±3.8	34.0±3.9	-
			Sample size (n)	29	13	-
			Intraoperative blood loss (mL)	2027.1±1637.6	3786.7±2936.1	<0.01
			Gestation (weeks)	34.8±1.5	35±2.2	<0.01
Bodner, 2006, [37]	USA	previa	Maternal age (years)	35.3	35.3	-
			Sample size (n)	6	22	-
			Intraoperative blood loss (mL)	2600	2800	0.4
			Blood transfusion (units)	6.3	6.5	0.47
			Gestation (weeks)	32.5	36.5	0.019
Picel, 2018, [38]	USA	previa	Sample size (n)	61	90	-
			Intraoperative blood loss (mL)	2000 (1500-2500)	2500 (2000-4000)	0.002
			Blood transfusion (units)	2 (0-5)	5 (2-8)	0.002
Wang, 2017, [39]	China	previa	Sample size (n)	10	43	-
			Intraoperative blood loss (mL)	1000	2000	<0.05
			Hysterectomy (%)	10	63.3	<0.05
Peng, 2020, [40]	China	previa	Maternal age (years)	32.08 ± 3.94	33.46 ± 4.53	-
			Sample size (n)	48	56	-
			Intraoperative blood loss (mL)	690.00 ± 226.88	887.10 ± 311.71	0.018
			Blood transfusion volume (ml)	970.54 ± 1083.21	1352.08 ± 1211.03	0.093
			Gestation (weeks)	35.57 ± 1.97	36.05 ± 1.66	0.177
			Hysterectomy (%)	12.5	29.2	0.035
El-sayed, 2016, [41]	Egypt	previa	Maternal age (years)	33.54±4.36	33.62± 3.86	-
			Sample size (n)	50	50	-
			Intraoperative blood loss (mL)	850±20	1120±70	0.000
			Blood transfusion (units)	1.8 ± 0.4	2.1 ± 0.5	0.001
			Hysterectomy (%)	10	12	-
Wu, 2016, [42]	China	previa	Maternal age (years)	29.5±3.6	30.4±4.0	-
			Sample size (n)	230	38	-
			Intraoperative blood loss (mL)	921±199	2790±335	0.000
			Blood transfusion volume (ml)	422±58	1580±67	0.000
			Gestation (weeks)	35.6±1.3	35.5±1.5	0.595
			Hysterectomy (%)	0.0	7.89	0.003
Feng, 2017, [43]	China	Accreta	Maternal age (years)	31.8±5.6	32.5±5.7	-
			Sample size (n)	30	11	-
			Intraoperative blood loss (mL)	1000(600-2500)	1100(800-2600)	0.64
			Blood transfusion (units)	0(0-6)	3(0-8)	0.67
			Gestation (weeks)	37 (36-37.28)	37.42(36.28-39)	0.069
Li, 2018, [44]	China	Accreta/Increta/Percreta	Maternal age (years)	34.53 ± 5.80	33.43 ± 4.32	-
			Sample size (n)	112	87	-
			Intraoperative blood loss (mL)	1850±490	3800±560	<0.001
			Blood transfusion volume (ml)	480±220	2000±1120	<0.001
			Gestation (weeks)	37.02 ± 1.3	36.78 ± 1.38	0.273
			Hysterectomy (%)	11.61	32.18	<0.001
Cali, 2014, [45]	Italy	Accreta/Increta/Percreta	Sample size (n)	30	23	-
			Intraoperative blood loss (mL)	846.67±280.06	1156.52±576.69	0.036
			Blood transfusion volume (ml)	470±860	1960±246	0.011
Dai, 2020, [46]	China	Accreta/Increta/Percreta	Maternal age (years)	34.08 ± 4.75	32.59 ± 4.10	-
			Sample size (n)	51	27	-
			Intraoperative blood loss (mL)	1846 ± 2187	3717 ± 2717	<0.001
			Blood transfusion (units)	5.65 ± 5.71	11.48 ± 8.72	<0.001
			Gestation (weeks)	34.94 ± 1.49	34.07 ± 4.19	0.178
			Hysterectomy (%)	2	11.1	0.081
Chou, 2015, [47]	Taiwan	Accreta/Increta/Percreta	Sample size (n)	13	11	-
			Intraoperative blood loss (mL)	1902.3±578.8	4445.7±996.48	0.0402
Zhou, 2019, [48]	China	Accreta/Increta/Percreta	Maternal age (years)	32.31 ± 4.20	32.52 ± 4.32	-
			Sample size (n)	58	25	-
			Intraoperative blood loss (mL)	1215.52 ± 762.57	1602.00 ± 862.47	0.045
			Blood transfusion volume (ml)	425.86 ± 667.43	832.80 ± 887.89	0.024
			Gestation (weeks)	35.95 ± 0.31	35.83 ± 0.34	0.144
			Hysterectomy (%)	6.9	12	0.445
Tan, 2017, [49]	Singapore	Accreta	Maternal age (years)	32 (27–37)	35 (29–43)	-
			Sample size (n)	11	14	-
			Intraoperative blood loss (mL)	2011 (400–5000)	3316 (1000–4000)	0.042
			Blood transfusion volume (ml)	1058 (0–3600)	2211 (1190–3980)	0.005
			Gestation (weeks)	36.2 (33–38)	35.7 (28.5–39)	-
McGinnis, 2019, [50]	Columbia	Accreta	Maternal age (years)	34 (24-44)	33 (26-37)	-
			Sample size (n)	12	12	-
			Intraoperative blood loss (mL)	2450 (1500-3125)	2750 (1875-3500)	0.28
			Blood transfusion (units)	3.5 (2.2-5.5)	9.5 (4.2-12)	0.06
			Gestation (weeks)	35 (27.9-37.7)	35 (27.9-37.7)	0.28
Panici, 2012, [51]	Italy	Accreta/Increta/Percreta	Maternal age (years)	29.4 ± 1.9	27.5 ± 1.7	-
			Sample size (n)	15	18	-
			Intraoperative blood loss (mL)	950(790-1100)	3375(2645-4007)	<0.001
			Blood transfusion (units)	0 (0-1)	4 (3-5)	<0.001
			Gestation (weeks)	36 ± 0.7	36 ± 0.6	-
			Hysterectomy (%)	13	50	0.034
Cho, 2020, [52]	Korea	Increta/Percreta	Maternal age (years)	34.5 ± 2.9	34.8 ± 3.3	-
			Sample size (n)	17	25	-
			Intraoperative blood loss (mL)	2319 (1000-4500)	4435 (1500-10500)	0.037
			Blood transfusion volume (ml)	2060 (1200-8000)	3840 (1800-15200)	0.043
			Hysterectomy (%)	29.4	44	0.339
Sun, 2018, [53]	China	Increta/Percreta	Sample size (n)	19	12	-
			Intraoperative blood loss (mL)	1200	3150	0.006
			Blood transfusion volume (ml)	800	1950	0.017
Zeng, 2017, [54]	China	Increta/Percreta	Maternal age (years)	32.27±5.27	33.13±5.23	-
			Sample size (n)	48	38	-
			Intraoperative blood loss (mL)	1467.71±1075.77	2218.42±1572.2	0.015
			Blood transfusion (units)	5.42±4.95	9.29±7.59	0.008
			Gestation (weeks)	36.04±2.18	35.41±2.42	0.205
			Hysterectomy (%)	4.2	23.7	0.018

### Intraoperative blood loss index (mL)

The first criterion for comparison was the intraoperative blood loss index (mL). In 26 studies [15, 20, 24, 26–40, 42–45, 47–50] prophylactic internal iliac artery balloon occlusion significantly reduced this index in the balloon occlusion group compared to the control group (*P* < 0.05). According to the results of studies by Yu et al. (2020) [9], Feng et al. (2017) [41] and McGinnis et al. (2019) [46], no significant difference was observed between the control group and the balloon occlusion group with respect to this index (*P* > 0.05).

**Fig. 2:**
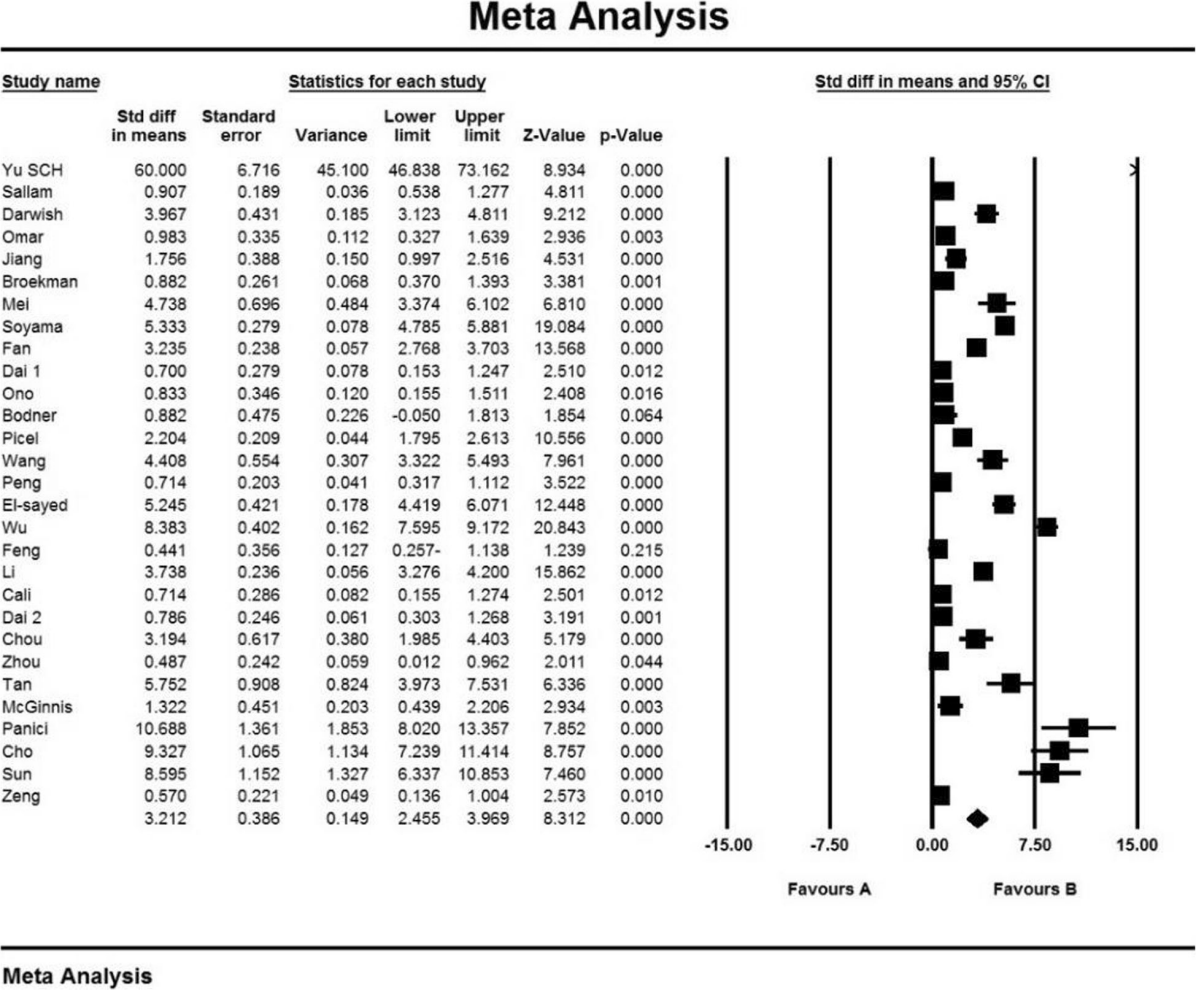
Accumulation diagram of the mean difference between case and control groups in intraoperative blood loss (mL) meta-analysis based on random model

**Fig. 3:**
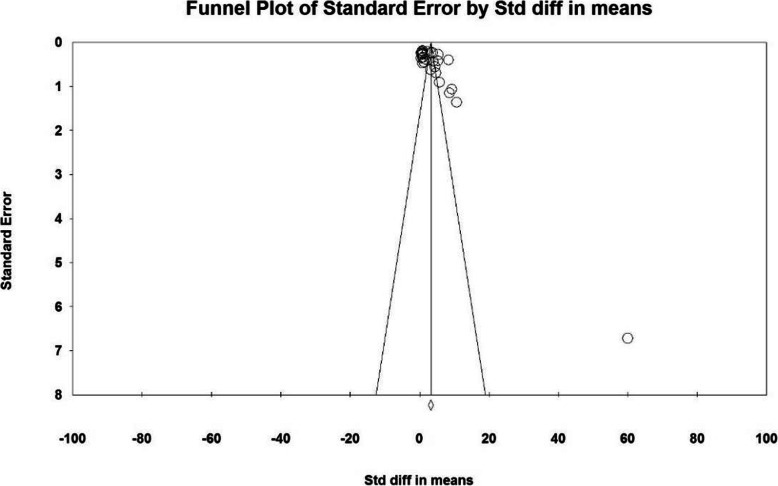
Funnel plot resulting from the difference between the mean of studies included in the Intraoperative blood loss (mL) meta-analysis in the case and control groups

Based on the results of I^2^ test, in the study of intraoperative blood loss index (mL), there was a heterogeneity between the control group and the case group (I^2^ = 97.4), thus, a random effects method was applied to amalgamate the results of all studies and approximate a final result. Based on the results of the meta-analysis, the mean difference of intraoperative blood loss (mL) in the balloon occlusion, and control groups were obtained as 1436 ± 211 and 403 ± 2503 respectively. Therefore, prophylactic internal iliac artery balloon occlusion reduces postpartum hemorrhage (Figs. 2 and 3). The graph of the mean difference between intraoperative blood loss (mL) in the case and control groups was 3.21 ± 0.38, which shows the positive effect of the intervention. The width of the horizontal line on each square denotes a 95 % confidence interval. Published bias was assessed using the Egger’s test, which showed no publication bias (*P* = 0.053).

### Blood transfusion index (units)

The second criterion is the comparison was blood transfusion index (units). In the studies of Dai et al. (2018) [34], Picel et al. (2018) [37], El-sayed et al. (2016) [39], Dai et al. (2020) [43], McGinnis et al. (2019) [46], Panici et al. (2012) [47] and Zeng et al. (2017) [50] significant differences between the control group and the balloon occlusion group in terms of blood transfusion index (units) ( *P* < 0.05) was observed. However, in reviewing the results of studies by Yu et al. (2020) [9], Darwish et al. (2014) [27], Omar et al. (2017) [28], Broekman et al. (2015) [30], Bodner et al. (2006) [36] and Feng et al. (2017) [41], no significant difference was observed between the control group and the balloon occlusion group in terms of this index (*P* > 0.05). Therefore, reduction of blood transfusion index (units) in the balloon occlusion group is not definite, and varies according to the study conditions. Thus, with respect to blood transfusion index (units), it can be argued that prophylactic internal iliac artery balloon occlusion has no advantage.

### Gestation index (weeks)

The third criterion for comparison was gestation index (weeks). In 14 studies [9, 15, 24, 26, 30, 32, 33, 40–43, 46, 47, 50] prophylactic internal iliac artery balloon occlusion did not significantly increased this index in the balloon occlusion group compared to the control group (P˃0.05), while in the study results of Darwish et al. (2014) [27], Ono et al. (2018) [35], Bodner et al. (2006) [36] and Tan et al. (2017) [45], there was a significant difference between the control group and the balloon occlusion group in terms of gestation index (weeks) (*P* < 0.05).

**Fig. 4:**
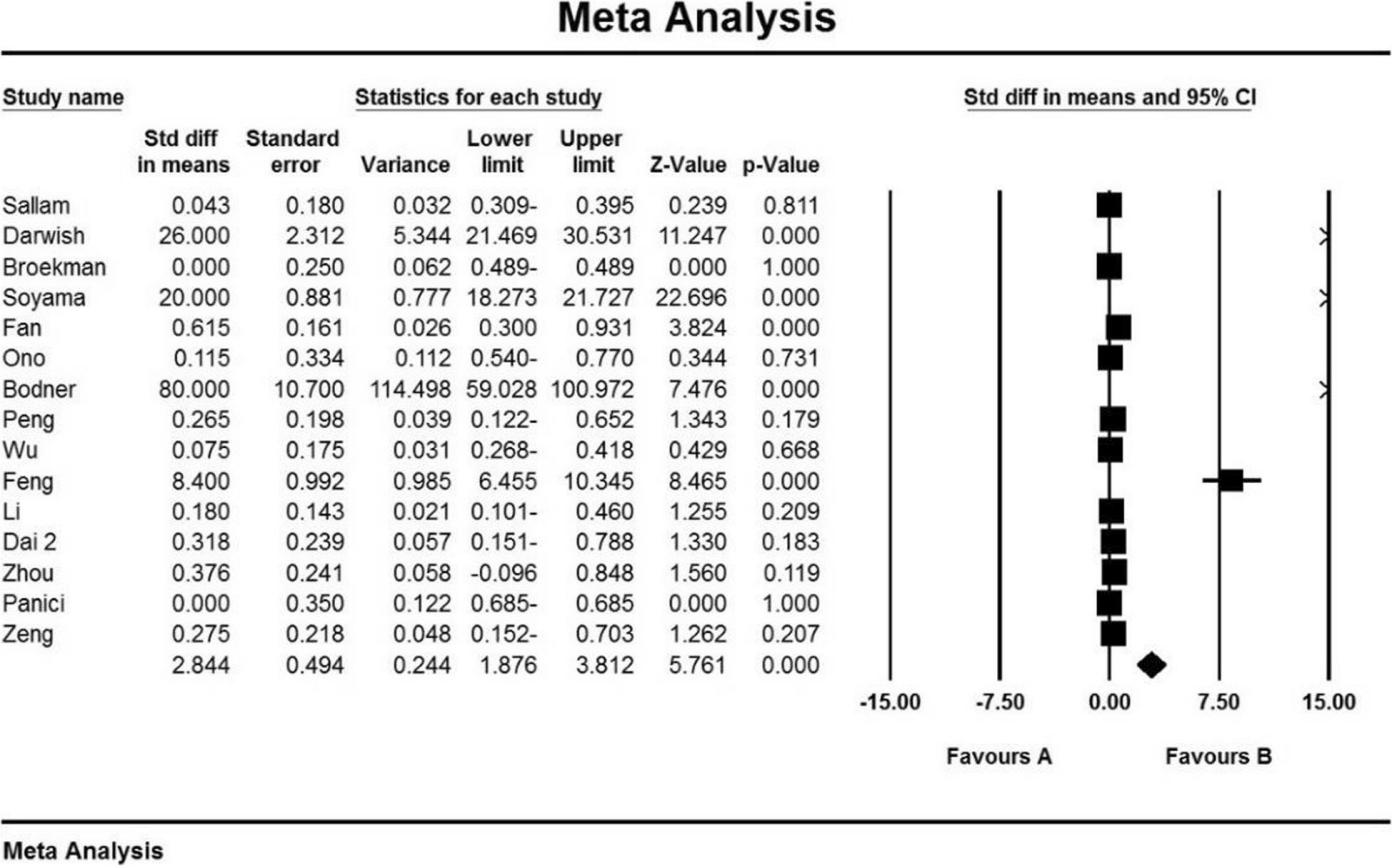
Accumulation diagram obtained from the difference between the mean of the studies included in the Gestation meta-analysis (weeks) in the case and control groups

**Fig. 5:**
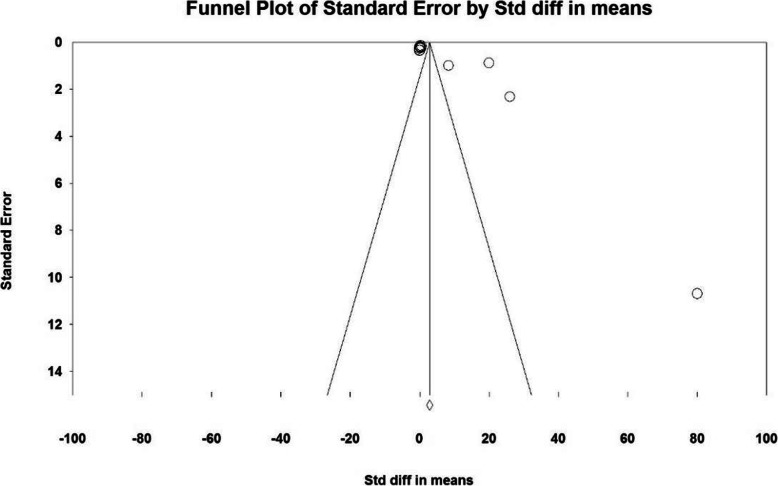
Funnel Plot resulting from the difference between the mean of the studies included in the Gestation (weeks) meta-analysis analysis in the case and control groups

Based on the results of I^2^ test, in the study of gestation index (weeks), there was heterogeneity between the control group (I^2^ = 99.1), thus, a random effects model was applied to combine the results of the studies and approximate a single final outcome. Based on the results of meta-analysis, mean of gestation (weeks) in the balloon occlusion and control groups were 37.0 ± 0.18 and 35.9 ± 0.17 respectively. Therefore, it can be concluded that prophylactic internal iliac artery balloon occlusion increases the gestation index (weeks). The mean difference between the two groups was 2.84 ± 0.49, which indicates the positive effect of the intervention. Figure 4 presents the forest plot for gestation (weeks) for different studies, as well as their confidence intervals. The horizontal line on each square also represents the 95 % confidence interval. Publication bias was assessed using the Egger’s test, which showed no bias in the studies (*P* = 0.055) (Figs. 4 and 5).

### Hysterectomy index (%)

The third criterion for comparison was the hysterectomy index (%). In 8 studies [24, 31, 34, 38, 40, 42, 47, 50] prophylactic internal iliac artery balloon occlusion significantly reduced this index in the balloon occlusion group compared to the control group (*P* < 0.05). While reviewing the results of studies by Omar et al. (2017) [28], Jiang et al. (2013) [29], Fan et al. (2017) [33], Dai et al. (2020) [43], Zhou et al. (2019) [15], and Cho et al. (2020) [48], no significant difference was observed between the control group and the balloon occlusion group in terms of hysterectomy index (%) (*P*˃0.05).

**Fig. 6:**
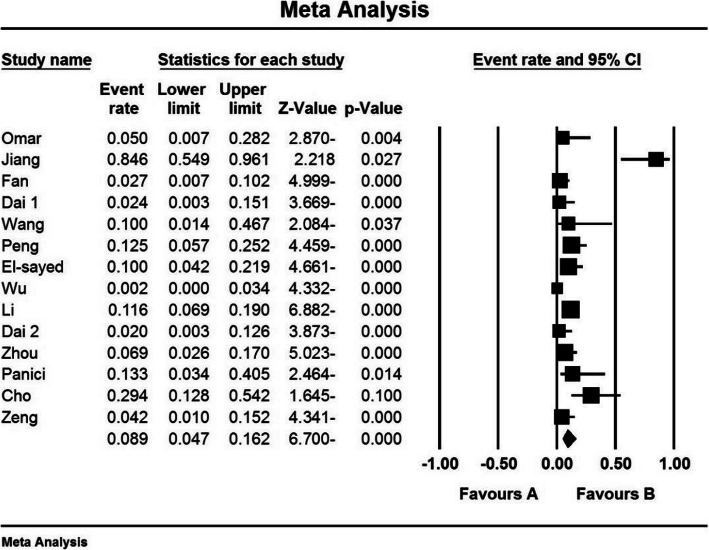
Accumulation diagram of the percentage of Hysterectomy studies included in the meta-analysis analysis in the case group

**Fig. 7:**
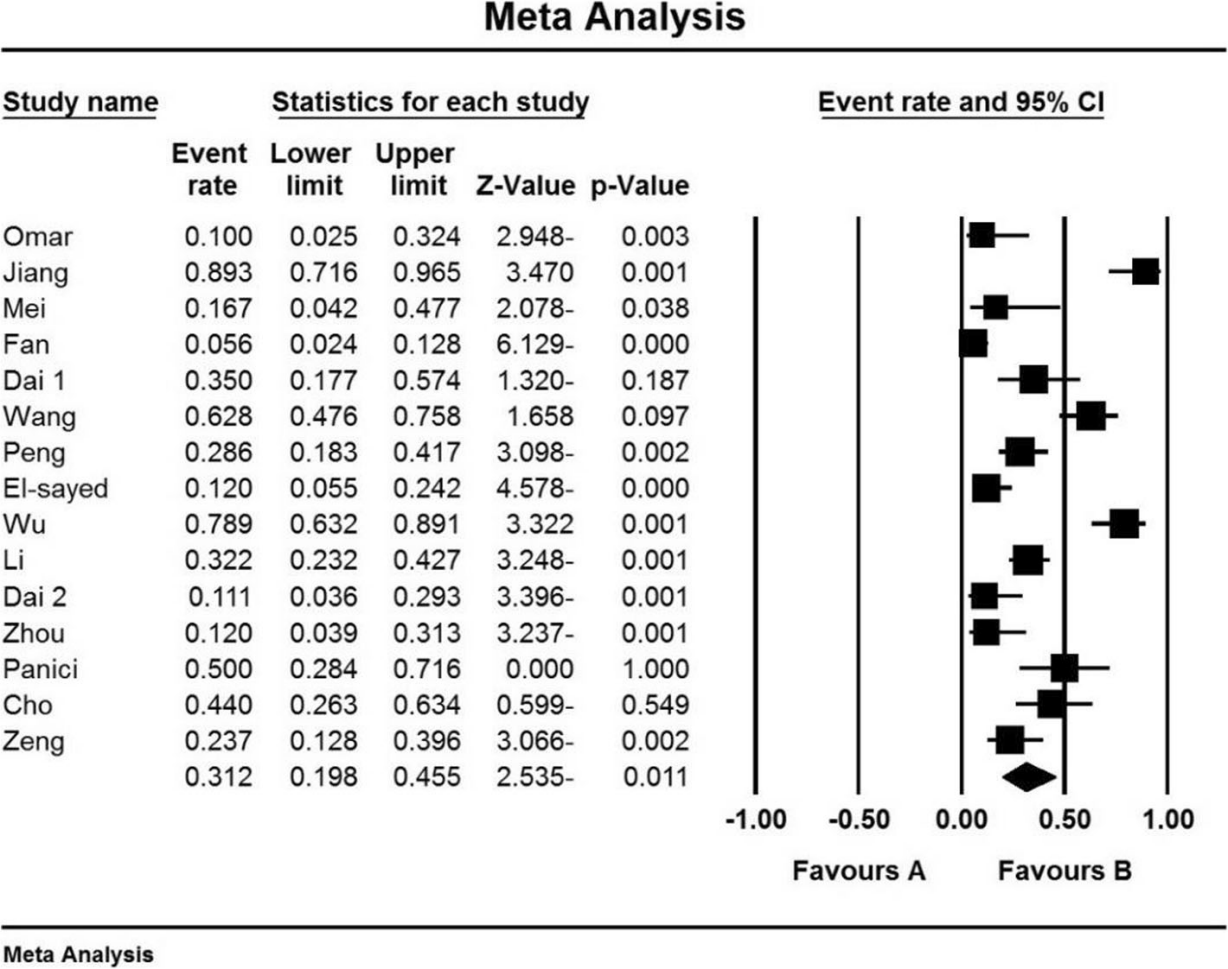
Accumulation diagram of Hysterectomy percentage of studies included in meta-analysis analysis in the control group

**Fig. 8:**
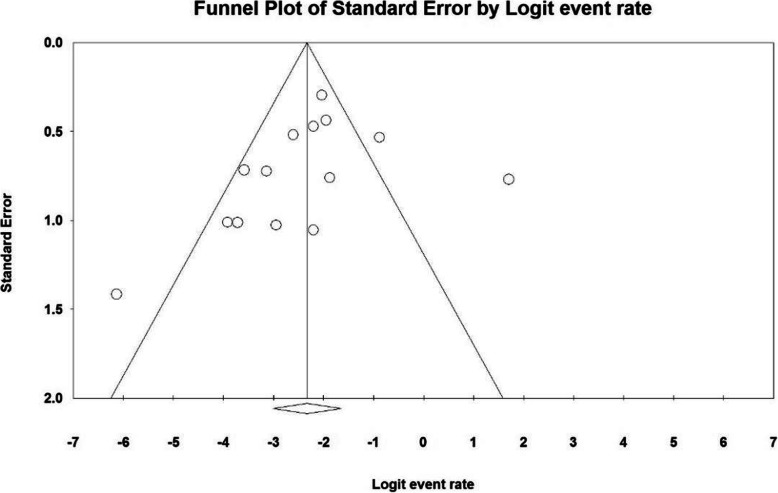
Funnel Plot resulting from the percentage of Hysterectomy studies included in the meta-analysis analysis in the case group

**Fig. 9:**
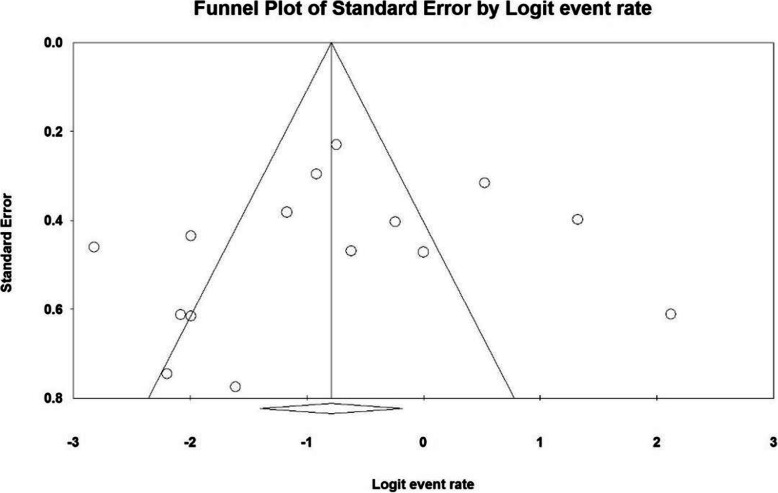
Funnel Plot resulting from the percentage of Hysterectomy studies included in the meta-analysis analysis in the control group

Based on the results of I^2^ test, in the study of hysterectomy index (%), there was a heterogeneity between the control group and the case group (I^2^ = 74.9), (I^2^ = 87.5), thus, a random effects method was applied to combine the results of the studies and approximate an overall. Based on the results of meta-analysis; the percentages of hysterectomy (%) in the balloon occlusion and control groups were 8.9 % and and 31.2 % respectively. Therefore, it can be concluded that prophylactic internal iliac artery balloon occlusion reduces the hysterectomy index (%), which indicates the positive effect of the intervention. The horizontal line on each square, in the hysterectomy forest plot, represents the 95 % confidence interval (Figs. 6 and 7). Publication bias was assessed using the Egger’s test, which shows no publication bias (*P* = 0.294) (*P* = 0.499) (Figs. 8 and 9).

## Discussion

The aim of the present systematic review and meta-analysis was to determine the effect of prophylactic internal iliac artery balloon occlusion in patients with placenta previa or Placenta accreta spectrum.

Bleeding is still a major cause of maternal death following childbirth. In many developed countries, bleeding is the most important factor in admitting a pregnant woman to an intensive care unit (ICU). In developing countries, bleeding is the cause of almost half of all maternal mortality [51]. Placenta previa is one of the causes of obstetric bleeding, which, by definition, is a placenta that covers the inner passageway of the cervix [51]. Placenta previa is a known risk factor for placenta accrete [52]; In a case-control study, placental bed biopsy was performed during cesarean section in 50 women with placenta previa and 50 cases with normal placenta. In approximately half of the placenta samples, spiral myometrium had infiltrating trophoblast giant cells, while only 20 % of samples related to normal pair, had such changes [53]. The placenta is an abnormal attachment of all or part of the placenta to uterine wall [54]. Due to the partial or complete absence of regular decidua and the incomplete development of the fibrinoid layer or the nitabuch membrane, the placental feathers stick to the myometrium. In the placenta increta, the villi enter the myometrium, and in the placenta increta, the villi invade the cervix or other pelvic organs. Different types of abnormal Placenta accreta spectrum are important causes of maternal mortality, due to bleeding during pregnancy or postpartum [51].

According to the present systematic review and meta-analysis, the mean difference of the two groups in terms of intraoperative blood loss (mL) was obtained 3.21 ± 0.38, and this difference was statistically significant. This also showed a positive effect of the intervention. The most valid study in terms of sample size, was a research conducted by Wu et al. (2016) [40], in which the authors reported that the intraoperative blood loss (mL) index in the case group was 921 ± 199 ml, whilst this 2790 ± 335 ml in the control group; the findings were statistically significant and consistent with the results of the present study.

Timely transfusion of blood and blood products can save the lives of these patients. Therefore, the use of a variety of blood products, can be prevent the risk of death in 15,000 pregnancies per year, globally [55]. Since blood reserves are sourced by donations, and this vital tissue requires different and sometimes rare blood types, proper management of blood transfusion can prevent surgery delays or even cancellations [56].

In 7 studies [34, 37, 39, 43, 46, 47, 50], a significant difference was reported between the control group and the balloon occlusion group in terms of blood transfusion index (units) (*P* < 0.05). However, in the results of 6 studies [9, 27, 28, 30, 36, 41], no significant difference was observed between the two groups (*P* > 0.05). According to the results results, the decrease in blood transfusion index (units) in the balloon occlusion group is not definite and varies according to the study conditions. Therefore, in terms of Blood transfusion index (units), it can be stated that prophylactic internal iliac artery balloon occlusion has no advantage.

Premature birth or gestational age less than 37 weeks from the last date of maternal menstruation is one of the most important indicators of health in any society, and the survival of newborns is directly related to gestational age and birth weight [57]. The prevalence of preterm birth varies in different societies, and statistics show that 8 to 10 % of infants born in the United States, and 5 to 7 % in Europe are born premature [58]. Various factors are involved in the birth of premature infants, including maternal, fetal, etc. [59]. Maternal causes such as maternal disease, chorioamnionitis, multiple births and maternal smoking, Placenta accreta spectrum, placenta previa, uterine defects, etc. can be mentioned [60].

According to the present systematic review and meta-analysis, the mean difference of the two groups in terms of gestation index (weeks) was 2.84 ± 0.49 which was statistically significant, and showed a positive effect of the intervention. The most valid study in terms of sample size was conducted by Wu et al. (2016) [40], which reported that the gestation index (weeks) in the case and control groups were 35.6 ± 1.3 and 35.5 ± 1.5 weeks respectively, which was not statistically significant; the results of the study was also not consistent with the general findings of the present work.

Hysterectomy is a major surgery that is almost always performed in cases of severe, life-threatening bleeding after vaginal delivery or cesarean section [61]. Its prevalence in the last century has been 1.4 ± 0.24 cases per 1000 live births [62]. Risk factors for emergency hysterectomy around delivery include abnormal Placenta adhesiens (percreta, increta, placenta accreta), placenta previa, uterine atony, previous cesarean section, and uterine rupture [63]. The most common cause reported in some studies was abnormal placental adherence with placenta previa in patients with a history of previous cesarean section [64, 65]. This surgery is associated with high maternal mortality even with modern midwifery [66]. Complications such as blood transfusion, fever, DIC and re-laparotomy in high rates, and maternal mortality have been reported in different studies [64, 65, 67].

According to the present systematic review and meta-analysis, the percentage of hysterectomy (%) in the balloon occlusion and hysterectomy control groups were 8.9 % and 31.2 % respectively, which was statistically significant and, showed a positive effect of the intervention. The most comprehensive study in terms of sample size was conducted by Wu et al. (2016) [40], who reported hysterectomy index (%) of 0 % in the case group and 7.89 % in the control group, which was statistically significant and is in line with the general findings of the present study.

In general, according to the present systematic review and meta-analysis; prophylactic internal iliac artery balloon occlusion in patients with placenta previa or Placenta accreta spectrum has benefits, such as reduced intraoperative blood loss, reduced hysterectomy, and increased gestation (weeks). Therefore, the procedure can be applied prophylactically by obstetricians and gynecologists.

One of the limitations of this study is that some samples were not based on random selection. Moreover, the non-uniform reporting of articles, and methods of implementation, lack of consistency in the data analysis, incomplete data, and the unavailability of the full text of some of the papers presented at the conferences can be mentioned.

## Conclusions

The results of this study show that prophylactic internal iliac artery balloon occlusion in patients with placenta previa or Placenta accreta spectrum has benefits such as reduced intraoperative blood loss, reduced hysterectomy and increased gestation (weeks), which can be considered by midwives and obstetricians.

## Data Availability

Datasets are available through the corresponding author upon reasonable request.

## Abbreviations

PAS: Placental accreta spectrum
STROBE: Strengthening the Reporting of Observational Studies in Epidemiology for cross- sectional Study
PRISMA: Preferred Reporting Items for Systematic Reviews and Meta-Analysis
